# Sex-Based Differences and Outcomes in Unselected Patients Undergoing Coronary Angiography

**DOI:** 10.3390/jcm14010224

**Published:** 2025-01-02

**Authors:** Lasse Kuhn, Tobias Schupp, Philipp Steinke, Kathrin Weidner, Thomas Bertsch, Jonas Rusnak, Mahboubeh Jannesari, Fabian Siegel, Daniel Duerschmied, Michael Behnes, Ibrahim Akin

**Affiliations:** 1 Department of Cardiology, Angiology, Haemostaseology and Medical Intensive Care, University Medical Centre Mannheim, Medical Faculty Mannheim, Heidelberg University, 68167 Mannheim, Germany; 2 Institute of Clinical Chemistry, Laboratory Medicine and Transfusion Medicine, Nuremberg General Hospital, Paracelsus Medical University, 90419 Nuremberg, Germany; 3 Department of Cardiology, Angiology and Pneumology, University Hospital Heidelberg, 69120 Heidelberg, Germany; 4 Department of Biomedical Informatics, Center for Preventive Medicine and Digital Health (CPD), Medical Faculty Mannheim, Heidelberg University, 68167 Mannheim, Germany

**Keywords:** sex, gender, coronary angiography, coronary revascularization, coronary artery disease, prognosis

## Abstract

**Background**: The study investigates sex-related differences and outcomes in unselected patients undergoing invasive coronary angiography (CA). Sex-based differences with regard to baseline characteristics and management of patients with cardiovascular disease have yet been demonstrated. However, their impact on long-term outcomes in unselected patients undergoing CA remains unknown. **Methods**: Consecutive patients undergoing invasive CA from 2016 to 2022 were included at one institution. Prognosis of male and female patients undergoing CA was investigated with regard to the primary endpoint of rehospitalization for heart failure (HF) at 36 months. Secondary endpoints comprised the risk of acute myocardial infarction (AMI) and coronary revascularization at 36 months, as well as in-hospital all-cause mortality. Statistical analyses included Kaplan–Meier analyses, as well as uni- and multivariable Cox proportional regression analyses. **Results**: From 2016 to 2022, 7691 patients undergoing CA were included (males: 65.1%; females: 34.9%). Males had a higher prevalence of coronary artery disease (CAD) (76.2% vs. 57.4%; *p* = 0.001), alongside a higher prevalence of 3-vessel CAD compared to females (33.9% vs. 20.3%; *p* = 0.001). The risk of rehospitalization for HF at 36 months was higher in males compared to females (22.4% vs. 20.3%; *p* = 0.036; HR = 1.127; 95% CI: 1.014–1.254; *p* = 0.027), which was no longer observed after multivariable adjustment. Male sex was associated with a higher risk of coronary revascularization (9.6% vs. 5.9%; *p* = 0.001; HR = 1.659; 95% CI: 1.379–1.997; *p* = 0.001), which was still evident after multivariable adjustment (HR = 1.650; 95% CI 1.341–2.029; *p* = 0.001). However, neither the risk of AMI at 36 months (8.1% vs. 6.9%; *p* = 0.077), nor the risk of in-hospital all-cause mortality (6.9% vs. 6.5%; *p* = 0.689) differed significantly between the two sexes. **Conclusions**: In consecutive patients undergoing coronary angiography, male sex was independently associated with an increased risk of coronary revascularization, but not HF-related rehospitalization.

## 1. Introduction

Cardiovascular diseases (CVDs) remain the leading cause of mortality, accounting for one third of all deaths worldwide [1]. One of the most important CVDs is coronary artery disease (CAD), affecting more than 7% of the adult population in the United States, with a higher prevalence in males compared to females [2]. The main factor in the development of CAD is atherosclerosis, which develops in a complex process of oxidation, inflammation, fibrosis, and calcification in the coronary artery wall, and ultimately leads to plaque formation [3,4]. The development of atherosclerosis is a prolonged process influenced by a variety of risk factors, such as hypercholesterolemia, hypertension, cigarette smoking, and diabetes mellitus [3,4]. Additionally, chronic kidney disease (CKD) is associated with increased morbidity and mortality related to CAD [5,6]. Male sex is acknowledged as risk factor for atherosclerosis, and males are at higher risk of developing CAD at young age compared to females [3,4]. Moreover, various studies suggested a higher prevalence of CAD, specifically multivessel disease, in males compared to females [7,8,9,10,11,12]. Nonetheless, there appears to be no significant or only minor differences in the frequency of revascularization procedures, such as percutaneous coronary intervention (PCI) or coronary artery bypass grafting (CABG), between males and females with stable coronary artery disease [13].

In prior studies, females undergoing invasive coronary angiography (CA) presented at higher age and with a higher burden of comorbidities, with heterogeneous findings regarding the risk of long-term mortality [7,14,15,16]. While some authors reported increased in-hospital mortality in females [7,14], others observed no sex-related differences with regard to short-term and long-term survival [15] or even mention female sex as an independent predictor for improved long-term survival in patients with stable CAD [16]. Even less data are available regarding the effect of sex on the risk of heart failure (HF) and coronary revascularization during long-term follow-up. Furthermore, comprehensive analyses investigating the prognostic impact of sex stratified by different indications for CA remains scarce.

The present study aims to investigate sex-based differences and outcomes in a large cohort of unselected patients undergoing CA. To address potential heterogeneity, the prognostic impact of sex was also analyzed across important subgroups, stratified by different indications for invasive CA.

## 2. Materials and Methods

### 2.1. Study Patients, Design, and Data Collection

This study included all consecutive and unselected patients who underwent CA at the University Medical Centre Mannheim (UMM), Germany, from January 2016 to August 2022. Operation and Procedure Classification (OPS) codes were used to identify the patients. Clinical data related to the index event were retrospectively collected using the local electronic hospital information system (SAP^®^, Walldorf, Germany). The collected data included symptoms, admission diagnoses, medical history, angiographic results, performed interventions, and discharge medications. Patients undergoing multiple CA procedures were included only once. The UMM includes a general emergency department that manages a wide range of conditions, including traumatic, surgical, neurological, and cardiovascular emergencies. Its cardiology department provides comprehensive services, including a 24-hour catheterization laboratory, an electrophysiology laboratory, a hybrid operating room, and telemetry units. Additionally, the UMM is integrated into a network of clinics that facilitate referrals for coronary artery bypass grafting (CABG) and other cardiac surgeries.

This research utilized data from a retrospective single-center registry comprising consecutive CA patients hospitalized at UMM (DRKS-ID: DRKS00034765). The registry was developed following the principles of the Declaration of Helsinki and was approved by the Medical Ethics Committee II of the Medical Faculty Mannheim, University of Heidelberg, Germany (ethics committee approval code: 2022-829; date of approval: 19 May 2022).

### 2.2. Inclusion and Exclusion Criteria

In this study, all consecutive patients aged 18 years or older who underwent invasive coronary angiography (CA) at the UMM were included. No additional exclusion criteria were applied. The procedures were conducted by interventional cardiologists following the latest European guidelines [17]. The decision to treat only the culprit lesion or all affected vessels was left to the discretion of the cardiologists performing the procedure. For this study, all source data from coronary angiographic procedures, including imaging files and reports, were retrospectively reviewed by two independent cardiologists. For the present study, the prognosis of male patients was compared to female patients.

### 2.3. Study Endpoints

In the present study, rehospitalization for HF at 36 months was assessed as the primary endpoint. Secondary endpoints included the risk of AMI at 36 months, the risk of coronary revascularization at 36 months, and in-hospital all-cause mortality. International Classification of Diseases (ICD) codes were used to identify all endpoints at the University Medical Centre Mannheim (UMM), Germany.

### 2.4. Statistical Methods

Quantitative data were reported as mean ± standard error of the mean (SEM), median with interquartile range (IQR), and full range, depending on the data distribution. Comparisons were conducted using Student’s *t*-test for normally distributed data or the Mann–Whitney U test for nonparametric data. The Kolmogorov–Smirnov test was applied to assess deviations from a Gaussian distribution. Qualitative data were expressed as absolute and relative frequencies and analyzed using the Chi-square test or Fisher’s exact test, as appropriate.

Kaplan–Meier analyses were performed to evaluate the risk of HF-related rehospitalization, AMI, and coronary revascularization, including all patients discharged alive. Univariable hazard ratios (HR) with 95% confidence intervals were calculated. Multivariable Cox regression models with the “forward selection” method were used to further examine the prognostic impact of sex. To assess the prognostic impact of sex stratified by different indications to perform CA, these analyses were conducted not only for the entire study cohort but also within predefined subgroups based on age (≥70 and <70 years), presence of angina, (non-)ST-elevation myocardial infarction ((N)STEMI), decompensated HF at index hospitalization, multivessel disease, and left ventricular ejection fraction (LVEF).

Statistical significance was defined as *p* ≤ 0.05 for all tests. Statistical analyses were performed using SPSS software (Version 25, IBM, Armonk, NY, USA).

## 3. Results

### 3.1. Study Population

From January 2016 to August 2022, a total of 7691 patients underwent invasive CA at the catheterization unit of the University Medical Centre Mannheim; 65.1% were males and 34.9% were females, respectively. Table 1 illustrates the distribution of patients’ characteristics and comorbidities. Males presented at a younger age than females (median 67 vs. 73 years; *p* = 0.001). The median body mass index (BMI) was higher in males compared to females (median 27.5 vs. 27.1 kg/m^2^; *p* = 0.001). There were no significant differences regarding the distribution of cardiovascular risk factors, such as arterial hypertension (85.2% vs. 85.2%; *p* = 0.971), diabetes mellitus (30.3% vs. 29.7%; *p* = 0.592), and hyperlipidemia (35.6% vs. 35.1%; *p* = 0.679). Males were more likely to have severely reduced LVEF (i.e., <35%) (17.5% vs. 10.5%; *p* = 0.001), whereas normal LVEF (i.e., >55%) was more prevalent in females (57.4% vs. 43.8%; *p* = 0.001). While unstable angina was more frequently observed in females (30.5% vs. 24.5%; *p* = 0.001), males were more frequently diagnosed with STEMI (13.6% vs. 8.5%; *p* = 0.001) or NSTEMI (19.0% vs. 16.2%; *p* = 0.003).

As shown in Table 2, the following differences between the two sexes were observed during index CA: males had a higher prevalence of CAD (76.2% vs. 57.4%; *p* = 0.001), alongside with a higher prevalence of 2-vessel (22.6% vs. 16.4%; *p* = 0.001) and 3-vessel disease (33.9% vs. 20.3%; *p* = 0.001). Consequently, PCI was more frequently performed in males than in females (47.1% vs. 35.5%; *p* = 0.001) and males were more frequently sent to CABG (5.5% vs. 2.3%; *p* = 0.001). Among the 3309 patients receiving PCI, 2329 patients (70.4%) received treatment of only one vessel, including 1638 (70.3%) males and 691 (29.7%) females. 980 patients (29.6%) received treatment of multiple vessels during index CA, of which 720 (73.5%) were male and 260 (26.5%) were female.

### 3.2. Prognostic Impact of Sex in Patients Undergoing CA

**Figure 1:**
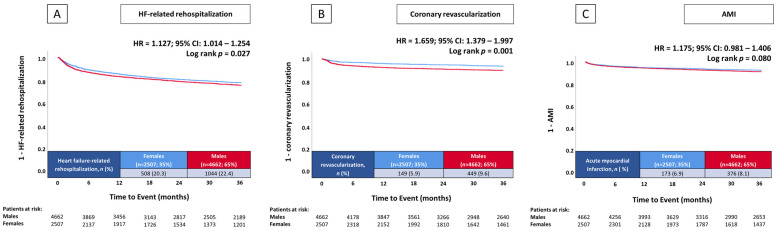
Prognostic impact of male sex in unselected patients on the risk of HF-related rehospitalization at 36 months (**A**), coronary revascularization at 36 months (**B**), and AMI at 36 months (**C**). AMI, acute myocardial infarction; CI, confidence interval; HF, heart failure; HR, hazard ratio.

The primary endpoint rehospitalization for HF at 36 months occurred in 22.4% of males and 20.3% of females (Figure 1). Males had a higher risk of rehospitalization for HF compared to females (HR = 1.127; 95% CI: 1.014–1.254; *p* = 0.027) (Figure 1). Additionally, coronary revascularization within 36 months was more frequently performed in males compared to females (9.6% vs. 5.9%, *p* = 0.001; HR = 1.659; 95% CI: 1.379–1.997; *p* = 0.001) (Figure 1). However, no significant sex-related differences were found with regard to the risk of rehospitalization for AMI at 36 months (8.1% vs. 6.9%, *p* = 0.077) (Figure 1) or in-hospital all-cause mortality (6.9% vs. 6.5%, *p* = 0.689) (Table 2).

### 3.3. Multivariable Cox Regression Analyses

After multivariable adjustment, male sex was no longer associated with significantly increased risk of rehospitalization for HF (HR = 1.067, 95% CI: 0.950–1.200; *p* = 0.274) (Table 3). However, patients’ age (HR = 1.009; 95% CI: 1.004–1.014; *p* = 0.001; per year increase), the presence of diabetes mellitus (HR = 1.233; 95% CI: 1.102–1.378; *p* = 0.001), prior CAD (HR = 1.552; 95% CI: 1.327–1.816; *p* = 0.001), prior CABG (HR = 1.237; 95% CI: 1.030–1.485; *p* = 0.023), atrial fibrillation (HR = 1.210; 95% CI: 1.078–1.357; *p* = 0.001), acute decompensated HF during index hospitalization (HR = 1.439; 95% CI: 1.263–1.639; *p* = 0.001), and LVEF < 35% (HR = 1.627; 95% CI: 1.551–1.707; *p* = 0.001) were associated with a higher risk of rehospitalization for HF at 36 months (Table 3). In contrast, a higher glomerular filtration rate (eGFR) (HR = 0.995; 95% CI: 0.993–0.998; *p* = 0.001; per 1 mL/min increase) and hemoglobin levels (HR = 0.944; 95% CI: 0.917–0.971; *p* = 0.001; per 1 g/dL increase) were associated with a lower risk of HF-related rehospitalization (Table 3).

With regard to secondary endpoints, male sex was associated with a 1.6-fold higher risk of coronary revascularization after multivariable adjustment (HR = 1.650, 95% CI: 1.341–2.029; *p* = 0.001) (Table 3).

In subgroup analyses, male sex was independently associated with a higher risk of coronary revascularization in patients both ≥70 years (HR = 1.660; 95% CI: 1.251–2.202; *p* = 0.001) and <70 years of age (HR = 1.584; 95% CI: 1.163–2.156; *p* = 0.004), as well as in patients presenting with unstable angina (HR = 1.555; 95% CI: 1.052–2.299; *p* = 0.027), STEMI (HR = 2.321; 95% CI: 1.215–4.432; *p* = 0.011), no evidence of CAD or single-vessel disease (HR = 1.986; 95% CI: 1.259–3.131; *p* = 0.003), and with LVEF ≥ 35% (HR = 1.626; 95% CI: 1.309–2.021; *p* = 0.001). In contrast, male sex had no significant effect on the risk of coronary revascularization at 36 months in other subgroups, neither in patients presenting with other CA indications, such as NSTEMI and acute decompensated HF on admission, nor in patients with multivessel disease and in patients with severely reduced LVEF (<35%) (Table 4).

## 4. Discussion

The present study aimed to investigate sex-based differences and outcomes in a large longitudinal cohort of consecutive and unselected patients undergoing CA in one single center. The main results of the study can be summarized as follows: males had a higher prevalence of CAD compared to females, alongside a higher prevalence of 3-vessel CAD and a subsequent higher need for coronary revascularization. The long-term risk of rehospitalization for HF was higher in males, which was no longer observed after multivariable adjustment. However, males were independently associated with a higher risk of coronary revascularization at 36 months, which was specifically seen in patients with unstable angina, STEMI, no/single-vessel CAD, and in patients with LVEF ≥ 35%.

The increased cardiovascular risk in males [2] may be attributed to various factors, including adverse lifestyle patterns [18,19], higher rates of abdominal obesity [20,21] and lower levels of the cardioprotective hormone estradiol [22,23]. From this perspective, decreasing levels of estradiol were demonstrated to contribute to an increase in cardiovascular risk in postmenopausal women and might explain the later onset of CAD in females than in males [2,3,24,25]. Consistent with this, the present study found that females undergoing CA were older compared to males. With increasing age, however, their cardiovascular risk appears to approach that of males, as no significant sex differences in the prevalence of cardiovascular risk factors, such as arterial hypertension, diabetes mellitus, and hyperlipidemia, were observed within the present study.

Nevertheless, sex-related differences can be found in several cardiovascular diseases. With regard to HF, the lifetime risk for males and females was shown to be comparable [26]. While HF is more prevalent in males among young and middle-aged patients, HF prevalence in females has been reported to surpass that in males in patients ≥80 years of age [2]. Sex differences become particularly apparent in the etiology, pathophysiology, and type of HF. While males are more prone to HF with reduced ejection fraction (HFrEF), females more commonly exhibit HF with preserved ejection fraction (HFpEF) [2,27]. Regarding HF etiology, ischemic causes play a greater role in males than in females [28]. Conversely, traditional risk factors such as diabetes mellitus, obesity, and hypertension represent stronger risk factors for the development of HF in females compared to males [29]. Differences in the prescription of HF pharmacotherapies between males and females may also affect the risk of rehospitalization for HF. In the present study, males were more frequently treated with ACE inhibitors (51.4% vs. 44.8%, *p* = 0.001), aldosterone antagonists (16.2% vs. 12.6%, *p* = 0.001), or SGLT2 inhibitors (5.5% vs. 3.6%, *p* = 0.001) compared to females, while the prescription of angiotensin receptor blockers was more frequent in females (28.2% vs. 21.6%, *p* = 0.001). The relatively low percentage of patients discharged with SGLT2 inhibitors is expected, considering the fact that patients were included from 2016 to 2022, while SGLT2 inhibitors were upgraded to a class 1A recommendation in 2021 at the end of the study period. In a similar period of time, similar prescription rates were observed in patients with HF and mildly reduced ejection fraction (HFmrEF) by our study group [30].

Aside from HF, sex differences are also evident in patients with CAD. Previous studies have established that not only is CAD prevalence higher in males compared to females [14] but also the rates of multivessel disease [9,31], multivessel PCI [7] and chronic total occlusion (CTO) [32,33]. In contrast, females are more prone to coronary microvascular disease and myocardial infarction with nonobstructive coronary arteries (MINOCA) [34,35,36]. In contrast to the present study, previous studies on the outcomes of CAD patients often included only patients with acute coronary syndrome (ACS) [37,38,39,40,41] or patients receiving PCI and/or CABG [7,31,37,39,42], and predominantly investigated the risk of mortality [14,31].

In line with our findings, the adjusted risk of in-hospital mortality in patients undergoing CA and/or PCI has been reported to be similar between males and females in older studies [43,44]. More recent studies, however, reported increased risk of in-hospital mortality for females even after multivariable adjustment [7,14].

With regard to major adverse cardiac events (MACE) following PCI, most commonly including cardiovascular death, AMI, and stroke, prior studies reported an apparent higher or similar risk in females compared to males [16,37,45]. The association of female sex with risk of AMI after PCI that was reported in other studies [39,45] was not confirmed in our study. Moreover, it remains uncertain whether the elevated risk of MACE in females may be entirely due to less favorable baseline characteristics [46,47,48]. Lee et al. reported a significantly higher adjusted long-term risk of death and MACE in males compared to females, including revascularization, shock, and stroke [48].

Rehospitalization for coronary revascularization or HF are events that should also be considered when evaluating the outcomes of patients undergoing CA. Meadows et al. observed that one third of ACS patients undergoing PCI were rehospitalized for cardiovascular events within 15 months. The majority (59.3%) of these cardiovascular event-related rehospitalizations were due to revascularization procedures, and only a small proportion of rehospitalizations were HF-related [38]. Regarding sex differences in rehospitalization for HF in STEMI patients, Zheng et al. found that females had higher rates of rehospitalization for HF compared to males; however, this difference was no more significant after multivariable adjustment [39], which is in line with the findings of the present study. Akyea et al. investigated outcomes in patients with coronary heart disease. During ten years of follow-up and after multivariable adjustment, males had a higher risk of recurrent coronary heart disease, which comprised admission for angina, myocardial infarction, PCI, or CABG [42]. Several other studies reported increased risk of revascularization after PCI in males, both in patients with acute and stable CAD [16,37,48].

In accordance with this, the present study found an increased adjusted risk of coronary revascularization at 36 months in males. In subgroup analyses, we observed that this difference is particularly evident in patients diagnosed with no obstructive CAD, single-vessel disease, a STEMI indication for CA, or a LVEF ≥ 35%. In patients with more severe and chronic conditions, i.e., patients with LVEF < 35%, decompensated HF on admission, or multivessel disease, no sex-related difference with regard to coronary revascularization at 36 months was observed. Male sex also had no significant impact on the risk of coronary revascularization at 36 months in patients undergoing CA for NSTEMI. Contrary to our findings, prior studies suggested that female STEMI patients had an increased adjusted mortality risk compared to males, while the opposite was true for NSTEMI patients [40,41,49,50]. Despite a lower mortality risk, Lin et al. reported that male AMI patients were at higher risk of recurrent revascularization [37], which in turn, supports the findings of the present study. Following the results of the present study, the prognostic impact of sex is more pronounced in CAD than in HF. As females are still underrepresented in many cardiovascular trials, they tend to receive less optimal therapy compared to males [51]. This may contribute to an ultimately similar prognosis between the two sexes regarding the progression of HF.

The prognostic impact of sex on the risk of coronary revascularization at 36 months was particularly evident in patients with STEMI and single-vessel disease, where a targeted therapy through culprit lesion PCI is possible. However, in high-risk patients with multivessel disease and/or severely reduced LVEF, there may be more important prognostic factors than sex.

### Study Limitations

The study has several limitations. Firstly, all data including follow-up data were sourced solely from hospital records. Patients’ prior medical history was assessed using OPS codes, which might lead to lower documented event rates of pre-existing illnesses. Because of the study’s retrospective design, explicit information on the indication for performing CA was not available. A major limitation of this study was the unavailability of data regarding long term all-cause mortality beyond index hospitalization—especially considering the risk interaction between rehospitalization and death. Additionally, the design of this study was retrospective and single-center. Therefore, results may be influenced by measured and unmeasured confounding factors, despite multivariable Cox regression analyses. Another limitation was the fact that all endpoints were assessed at our institution only. Results may not be generalizable to broader populations in different countries outside of Germany.

## 5. Conclusions

In unselected patients undergoing CA, male sex was independently associated with increased risk of coronary revascularization at 36 months, whereas the risk of HF-related rehospitalization was comparable to female sex after multivariable adjustment. This was particularly evident in patients with STEMI, no evidence of CAD, single-vessel disease, or LVEF ≥ 35%.

## Figures and Tables

**Table 1:** Baseline characteristics.

	Males (*n* = 5009)		Females (*n* = 2682)		*p* Value
**Age**, median (IQR)	67	(57–77)	73	(63–80)	**0.001**
**Body mass index**, kg/m^2^, median (IQR)	27.5	(24.7–30.8)	27.1	(23.6–31.6)	**0.001**
**Cardiovascular risk factors**, *n* (%)					
Arterial hypertension	4266	(85.2)	2285	(85.2)	0.971
Diabetes mellitus	1518	(30.3)	797	(29.7)	0.592
Hyperlipidemia	1783	(35.6)	942	(35.1)	0.679
**Prior medical history**, *n* (%)					
Pacemaker	99	(2.0)	17	(0.6)	**0.001**
COPD	185	(3.7)	112	(4.2)	0.295
Chronic kidney disease	252	(5.0)	172	(6.5)	**0.011**
Liver cirrhosis	57	(1.1)	32	(1.2)	0.829
Malignancy	285	(5.7)	161	(6.0)	0.575
Stroke	39	(0.8)	21	(0.8)	0.983
**Comorbidities at index hospitalization**, *n* (%)					
Acute coronary syndrome					
Unstable angina	1225	(24.5)	819	(30.5)	**0.001**
STEMI	683	(13.6)	228	(8.5)	**0.001**
NSTEMI	951	(19.0)	435	(16.2)	**0.003**
Atrial fibrillation	1237	(24.7)	778	(29.0)	**0.001**
Atrial flutter	112	(2.2)	50	(1.9)	0.279
Acute decompensated heart failure	585	(11.7)	342	(12.8)	0.169
Cardiogenic shock	230	(4.6)	92	(3.4)	**0.015**
Atrioventricular block	134	(2.7)	61	(2.3)	0.287
Cardiopulmonary resuscitation					
Out-of-hospital	282	(5.6)	104	(3.9)	**0.001**
In-hospital	120	(2.4)	47	(1.8)	0.065
Valvular heart disease	785	(15.7)	517	(19.3)	**0.001**
Stroke	185	(3.7)	103	(3.8)	0.746
**LVEF**, *n* (%)					
>55	1964	(43.8)	1379	(57.4)	**0.001**
45–55%	1063	(23.7)	478	(19.9)	
35–44%	674	(15.0)	292	(12.2)	
<35%	785	(17.5)	253	(10.5)	
Not documented	523		280		

COPD, chronic obstructive pulmonary disease; LVEF, left ventricular ejection fraction; IQR, interquartile range; (N)STEMI, (non)-ST-segment elevation myocardial infarction. Level of significance *p* ≤ 0.05. Bold type indicates statistical significance.

**Table 2:** Procedural, laboratory and follow-up data.

	Male (*n* = 5009)		Female (*n* = 2682)		*p* Value	
**Coronary angiography,***n* (%)						
No evidence of coronary artery disease	1194	(23.8)	1142	(42.6)	**0.001**	
1-vessel disease	981	(19.6)	554	(20.7)		
2-vessel disease	1134	(22.6)	441	(16.4)		
3-vessel disease	1700	(33.9)	545	(20.3)		
Right coronary artery	2622	(52.3)	962	(35.8)	**0.001**	
Left main trunk	648	(12.9)	198	(7.4)	**0.001**	
Left anterior descending	3019	(60.3)	1162	(43.3)	**0.001**	
Left circumflex	2383	(47.6)	820	(30.6)	**0.001**	
Ramus intermedius	667	(13.3)	183	(6.8)	**0.001**	
CABG	193	(3.9)	34	(1.3)	**0.001**	
Chronic total occlusion	489	(9.8)	127	(4.7)	**0.001**	
**PCI**, *n* (%)	2358	(47.1)	951	(35.5)	**0.001**	
Right coronary artery	928	(18.5)	359	(13.4)	**0.001**	
Left main trunk	198	(4.0)	94	(3.5)	0.327	
Left anterior descending	1221	(24.4)	506	(18.8)	**0.001**	
Left circumflex	807	(16.1)	286	(10.7)	**0.001**	
Ramus intermedius	105	(2.1)	32	(1.2)	**0.004**	
CABG	44	(0.9)	11	(0.4)	**0.020**	
**Sent to CABG**, *n* (%)	277	(5.5)	62	(2.3)	**0.001**	
**Procedural data**						
Number of stents, median (IQR)	2	(1–3)	2	(1–3)	**0.001**	
Stent length, median (IQR)	44	(24–78)	40	(20–68)	**0.001**	
Contrast, mL, median (IQR)	126	(76–207)	97	(76–167)	**0.001**	
**Baseline laboratory values**, median (IQR)						
Sodium, mmol/L	139	(138–141)	140	(138–141)	0.327	
Potassium, mmol/L	3.99	(3.76–4.22)	3.90	(3.67–4.15)	**0.001**	
Calcium, mmol/L	2.20	(2.12–2.29)	2.22	(2.13–2.31)	**0.001**	
Creatinine, mg/dL	1.07	(0.92–1.38)	0.92	(0.78–1.21)	**0.001**	
eGFR, mL/min/1.73 m^2^	72	(54–87)	63	(47–80)	**0.001**	
Urea, mg/dL	38.2	(30.1–53.4)	37.6	(28.6–52.4)	**0.001**	
Hemoglobin, g/dL	13.7	(12.1–14.9)	12.5	(11.1–13.6)	**0.001**	
WBC count, ×10^9^/L	8.95	(7.16–11.44)	8.90	(7.08–11.11)	0.093	
Platelet count, ×10^9^/L	226	(184–272)	252	(209–301)	**0.001**	
HbA1c, %	5.8	(5.5–6.6)	5.8	(5.5–6.6)	0.758	
LDL-cholesterol, mmol/L	103	(77–134)	107	(82–137)	**0.001**	
HDL-cholesterol, mmol/L	40.0	(33.0–48.0)	50	(39.3–48.0)	**0.001**	
Triglycerides, mg/dL	126	(94–178)	127	(94–174)	0.753	
C-reactive protein, mg/L	27	(9–86)	24	(8–76)	0.152	
Procalcitonin, µg/L	0.43	(0.13–1.92)	0.30	(0.11–1.50)	**0.030**	
Albumin, g/L	34.55	(30.70–37.45)	33.80	(30.05–36.65)	**0.001**	
INR	1.1	(1.0–1.2)	1.1	(1.0–1.1)	**0.001**	
NT-pro BNP, pg/mL	1899	(460–5602)	2311	(631–6093)	**0.002**	
Cardiac troponin I, µg/L	0.82	(0.12–7.41)	0.55	(0.11–3.29)	**0.001**	
Creatin Kinase, U/L	153	(92–360)	107	(68–206)	**0.001**	
Creatin Kinase MB, U/L	32	(21–68)	21	(20–59)	**0.004**	
**Medication at discharge**, *n* (%)						
ACE-inhibitor	2522	(51.4)	1123	(44.8)	**0.001**	
ARB	1008	(21.6)	708	(28.2)	**0.001**	
Beta-blocker	3323	(71.3)	1750	(69.8)	0.187	
Aldosterone antagonist	757	(16.2)	316	(12.6)	**0.001**	
ARNI	57	(1.2)	21	(0.8)	0.134	
SGLT2-inhibitor	256	(5.5)	91	(3.6)	**0.001**	
Statin	3574	(76.6)	1720	(68.6)	**0.001**	
ASA	3200	(68.6)	1435	(57.2)	**0.001**	
P2Y12-inhibitor	2412	(51.8)	989	(39.4)	**0.001**	
OAC	1211	(26.0)	787	(31.4)	**0.001**	
**Follow-up data**, median (IQR)						
Hospitalization time	7	(4–12)	7	(4–13)	**0.001**	
**All-cause mortality, in hospital**, *n* (%)	3467	(6.9)	175	(6.5)	0.689	
**Patients discharged alive**, *n* (%)	4663	(93.1)	2507	(93.5)		
**Primary endpoint**, *n* (%)						
HF-related rehospitalization, at 36 months	1044	(22.4)	508	(20.3)	**0.036**	
**Secondary endpoints**, *n* (%)						
Coronary revascularization, at 36 months	449	(9.6)	149	(5.9)	**0.001**	
Acute myocardial infarction, at 36 months	376	(8.1)	173	(6.9)	0.077	

ACE, angiotensin-converting enzyme; ARB, angiotensin receptor blocker; ARNI, angiotensin receptor neprilysin inhibitor; ASA, acetylsalicylic acid; CABG, coronary artery bypass grafting; eGFR, estimated glomerular filtration rate; HbA1c, glycated hemoglobin; HDL, high-density lipoprotein; INR, international normalized ratio; IQR, interquartile range; LDL, low-density lipoprotein; NT-pro BNP, aminoterminal pro-B-type natriuretic peptide; OAC, oral anticoagulant; PCI, percutaneous coronary intervention; SGLT2, sodium glucose linked transporter 2; WBC, white blood cells. Level of significance *p* ≤ 0.05. Bold type indicates statistical significance.

**Table 3:** Multivariable Cox regression analyses with regard to the risk of HF-related rehospitalization at 36 months, coronary revascularization at 36 months, and AMI at 36 months.

Variable	HF-Related Rehospitalization		Coronary Revascularization		AMI	
	HR (95% CI)	*p* Value	HR (95% CI)	*p* Value	HR (95% CI)	*p* Value
Age	1.009 (1.004–1.014)	**0.001**	1.001 (0.993–1.009)	0.875	0.986 (0.978–0.994)	**0.001**
BMI	1.009 (0.999–1.019)	0.078	1.009 (0.993–1.025)	0.283	0.995 (0.979–1.011)	0.514
DM	1.233 (1.102–1.378)	**0.001**	1.485 (1.237–1.782)	**0.001**	1.277 (1.059–1.541)	**0.011**
Prior CAD	1.552 (1.327–1.816)	**0.001**	1.496 (1.151–1.945)	**0.003**	1.186 (0.894–1.575)	0.237
Prior CABG	1.237 (1.030–1.485)	**0.023**	0.981 (0.692–1.392)	0.915	1.118 (0.804–1.555)	0.506
Prior MI	0.996 (0.738–1.345)	0.980	0.892 (0.491–1.618)	0.706	1.547 (0.969–2.467)	0.067
STEMI	0.988 (0.817–1.196)	0.902	1.865 (1.452–2.394)	**0.001**	1.064 (0.791–1.432)	0.681
NSTEMI	0.911 (0.791–1.049)	0.194	1.715 (1.402–2.098)	**0.001**	1.659 (1.348–2.042)	**0.001**
AF	1.210 (1.078–1.357)	**0.001**	0.858 (0.691–1.065)	0.165	1.002 (0.816–1.230)	0.988
Decompensated HF	1.439 (1.263–1.639)	**0.001**	0.783 (0.582–1.052)	0.104	1.370 (1.094–1.717)	**0.006**
LVEF	1.627 (1.551–1.707)	**0.001**	1.027 (0.944–1.118)	0.537	1.693 (1.563–1.834)	**0.001**
eGFR	0.995 (0.993–0.998)	**0.001**	1.002 (0.998–1.005)	0.391	0.998 (0.995–1.002)	0.418
Hemoglobin	0.944 (0.917–0.971)	**0.001**	0.962 (0.917–1.008)	0.103	0.979 (0.934–1.027)	0.380
Male sex	1.067 (0.950–1.200)	0.274	1.650 (1.341–2.029)	**0.001**	0.958 (0.786–1.167)	0.668

AF, atrial fibrillation; AMI, acute myocardial infarction; BMI, body mass index; CABG, coronary artery bypass grafting; CAD, coronary artery disease; CI, confidence interval; DM, diabetes mellitus; eGFR, estimated glomerular filtration rate; HF, heart failure; HR, hazard ratio; LVEF, left ventricular ejection fraction; MI, myocardial infarction; (N)STEMI, (non-)ST elevation myocardial infarction. Level of significance *p* ≤ 0.05. Bold type indicates statistical significance.

**Table 4:** Prognostic impact of male vs. female sex with regard to the risk coronary revascularization at 36 months in different subgroups.

Subgroup	Coronary Revascularization	
	HR (95% CI)	*p* Value
Age ≥ 70	1.660 (1.251–2.202)	**0.001**
Age < 70	1.584 (1.163–2.156)	**0.004**
Angina	1.555 (1.052–2.299)	**0.027**
STEMI	2.321 (1.215–4.432)	**0.011**
NSTEMI	1.213 (0.824–1.785)	0.327
Decompensated HF	1.447 (0.800–2.619)	0.222
0-/1-vessel disease	1.986 (1.259–3.131)	**0.003**
Multivessel disease	1.085 (0.859–1.371)	0.493
LVEF ≥ 35%	1.626 (1.309–2.021)	**0.001**
LVEF < 35%	1.744 (0.870–3.498)	0.117

Multivariable analyses in different subgroups were adjusted for age, BMI, diabetes mellitus, prior coronary artery disease, prior coronary artery bypass grafting, prior myocardial infarction, STEMI, NSTEMI, atrial fibrillation, decompensated HF at index hospitalization, LVEF, eGFR and hemoglobin. CI, confidence interval; HF, heart failure; HR, hazard ratio; LVEF, left ventricular ejection fraction; (N)STEMI, (non-)ST elevation myocardial infarction. Level of significance *p* ≤ 0.05. Bold type indicates statistical significance.

## Data Availability

The datasets used and/or analyzed during the current study are available from the corresponding author upon reasonable request.
